# Depression in Hospitalised Aphasic Stroke Patients—Identifying Valid Tools and Examining Outcomes

**DOI:** 10.3390/jcm15093187

**Published:** 2026-04-22

**Authors:** Zhi Qi Nicole Lim, Lai Gwen Chan

**Affiliations:** 1 Lee Kong Chian School of Medicine, NTU Singapore, Singapore 308232, Singapore; 2 Psychiatry, Tan Tock Seng Hospital, Singapore 308433, Singapore

**Keywords:** neuropsychiatry, depression, anxiety, aphasia, stroke, outcomes

## Abstract

**Objectives:** This study aimed to assess the validity of aphasic depression and anxiety rating scales (Signs of Depression Scale [SODS], Hospital version of Stroke Aphasic Depression Questionnaire [SADQH-10], Behavioural Outcomes of Ansxiety scale [BOA]) in the local setting and describe clinical outcomes of a local aphasic stroke population. **Methods**: Records of 236 aphasic stroke patients from an existing database of a proactive post-stroke depression screening service in a tertiary stroke centre in Singapore with rehabilitation facilities met review criteria for having available data for the SODS, SADQH-10 and carer-rated version of BOA (BOA-C). The reference standard was a psychiatrist’s diagnosis of post-stroke mood disorder. Clinical outcomes at 1-year post-stroke were analysed. **Results**: The areas under the Receiver Operator Characteristic curve (AUC) compared against the psychiatrist’s diagnosis of post-stroke mood disorder were 0.805, 0.844 and 0.780 for the SODS, SADQH-10 and BOA-C respectively. Optimal cut-off scores were found for the SODS (four and above) and SADQH (seven and above), with a sensitivity of 60.00% and 55.56%, respectively, and a specificity of 94.62% and 96.17%, respectively. No appropriate cut-off score was found for the BOA-C. Of the patients, 58.6% were found to have poor outcomes at 1-year post-stroke, and recurrent and haemorrhagic strokes were significant predictors of poor outcomes. **Conclusions**: The SODS and SADQH-10 are appropriate tools for assessing post-stroke depression in hospitalised aphasic stroke patients in Singapore based on their predictive values and likelihood ratios. The BOA-C has poor validity. At 1-year post-stroke, more than 50% of aphasic stroke patients have poor clinical outcomes not associated with clinician-diagnosed depression status, but predicted by recurrent and haemorrhagic strokes.

## 1. Introduction

In the acute phase of stroke, up to 33% of stroke survivors have aphasia [1], which is an acquired language disability affecting the comprehension and/or expression of language [2] and a common neuropsychological stroke sequelae [3]. In the sub-acute phase, up to 18% of stroke survivors remain aphasic [1]. Aphasia has been found to be associated with increased mortality and significant adverse effects on functional, occupational recovery and psychological wellbeing [4].

Post-stroke depression (PSD) affects at least one third of stroke survivors internationally [5], and only about 5% of these patients are identified and treated [6]. The pathophysiology and mechanism of PSD is multifactorial—from physical insult and changes in neurobiological pathways to the significant psychosocial impact of stroke [7]. Risk factors for PSD include size of stroke lesion, location of stroke, proximity of stroke lesion to the frontal lobe [8], previous history of depression [9], and a lack of psychosocial support [3]. PSD negatively impacts quality of life, and more importantly, increases the risk of stroke recurrence and death [10]. It is typically screened for by self-report questionnaires and diagnosed by clinical interview according to the DSM-V (Diagnostic and Statistical Manual) criteria [11].

Previous research shows that aphasic stroke patients are also more likely to suffer from PSD compared to those that do not have aphasia [12]. However, aphasic stroke patients are unable to be assessed with the usual screening and diagnostic methods, as their impaired comprehension and expression of language prohibits appropriate verbal and non-verbal responses in a clinical interview. Signs of PSD are also confounded by neurological sequelae such as psychomotor slowing, fatigue, apathy, and cognitive impairment. Without timely and precise diagnosis and treatment of PSD, these patients are at risk of poor participation in early neurorehabilitation, which will impede stroke and aphasia recovery. Similarly, more than 50% of stroke survivors experience anxiety within ten years, and most of such patients have anxiety symptoms as early as three months post-stroke [13]. Despite its prevalence, post-stroke anxiety (PSA) and its relevant clinical impact remains much less explored compared to PSD [14]. Moreover, anxiety symptoms are often co-occurring with depressive symptoms in PSD [15] and are potentially useful in helping to distinguish PSD from other post-stroke neurological sequelae.

Hence, to aid in the timely identification of mood disorders in aphasic stroke survivors, there has been the development of carer-rated aphasic depression rating scales, such as the Hospital version of Stroke Aphasic Depression Questionnaire (SADQH-10), the Signs of Depression Scale (SODS), and a carer-rated aphasic anxiety scale, the Behavioural Outcomes of Anxiety scale (carer version) (BOA-C), which are based on observable behavioural manifestations of depression and anxiety. However, validation of these tools has been scarce and challenging due to the impaired verbal abilities of this patient group, and none have been performed in the local setting. A systematic review by Rose et al. in 2023 showed that such validation studies had varying methodological quality largely due to differing reference standards and heterogenous and small samples [16]. In particular, the authors did not find any self-report measures that could be recommended, hence concluding that clinician and observer-rated measures are more appropriate [16].

This study aims to validate the SODS, SADQH-10 and BOA-C in our local population against a psychiatrist’s clinical diagnosis of mood disorder post-stroke as the reference standard. These tools were chosen due to qualitative feedback from relevant healthcare professionals on the feasibility, acceptability, and face validity. A secondary objective of the study is to examine the outcomes of aphasic stroke patients after 1 year, as we hypothesise that more than half of the local aphasic stroke patients will have poor outcomes and that PSD will be predictive.

## 2. Materials and Methods

A retrospective record review was performed for consecutive aphasic post-stroke patients who were referred to a proactive post-stroke depression screening service in a tertiary stroke centre with rehabilitation facilities in Singapore from 1 August 2021 to 30 June 2024. This psychiatry service routinely screens referred acute stroke patients for psychiatric comorbidity and sequelae [17]. Screening is conducted by trained allied health professionals with a battery of tools, administered to carers or survivors and supervised by a Consultant Neuropsychiatrist. The Consultant Neuropsychiatrist then performs a clinical review after the screening using multiple sources of information in correlation with the screening outcome. The battery of tools administered to aphasic stroke patients include the SODS, SADQH-10, and BOA (either carer or survivor version).

This study was approved by the Domain Specific Review Boards (DSRBs) of the National Healthcare Group.

### 2.1. Inclusion and Exclusion

**Figure 1:**
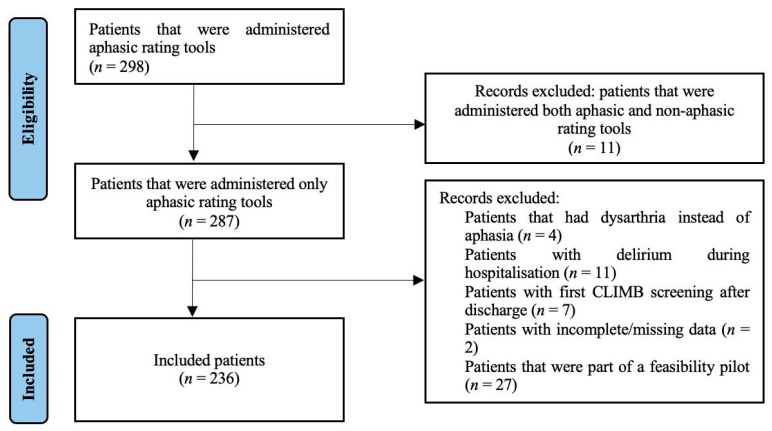
Record selection flowchart.

The records of all aphasic stroke patients were eligible for review. Records were excluded for reasons including the inability to perform screening due to delirium or other medical reasons or if there was too much missing data, as seen in Figure 1. Amongst the 236 cases included there were 145 SODS responses and 206 BOA-C responses, resulting in different sample sizes for each screening tool.

### 2.2. Baseline Demographics and Clinical Characteristics

The demographic variables and clinical characteristics of suitable patients were extracted from clinical notes and the screening service’s standing database. These include the demographic data (age, sex, race, marital status, highest educational level, occupation), clinical information (clinician’s diagnosis, clinical outcomes, antidepressant prescription) and stroke characteristics (laterality, incidence, nature, type, circulation, special structures).

### 2.3. Outcome Measures

The SODS is a carer-rated 6-item questionnaire, with each item dichotomously rated ‘yes’, giving a score of 1, or ‘no’, giving a score of 0. The total score ranges from 0 to 6, with a higher score representing more distress. It was originally developed for elderly hospitalised patients and is recommended for its ease in daily clinical usage [18]. In a validation study by van Dijk et al., the tool showed good internal consistency and criterion validity using a clinical interview as a reference standard [19].

The SADQ-Hospital-10 (SADQH-10) is a carer-rated 10-item version of the SADQ-Hospital (SADQH), a 21-item questionnaire developed based on behaviours that reflect depressed mood [20] for use with hospitalised patients. Recommended for its inpatient application where the patient’s behaviour in the past week is rated by the nurse or healthcare practitioner, the items in SADQH-10 each have a score ranging from 0 to 3, giving a total score within the range of 0–30. Higher scores represent a higher frequency of symptoms and increased distress. As compared to the SADQ-10, a 10-item questionnaire that is suitable for clinical use not specifically confined to the hospital, the SADQH-10 has rephrased items that are more catered towards patients with aphasia and disability. Bennett et al. found the internal consistency of SADQH-10 to be acceptable (α = 0.77) [21].

The BOA-C is a 10-item carer-rated questionnaire developed from psychologists’ experience and the literature for anxiety screening in patients with cognitive and communication impairments. Each item has a score ranging from 0 to 3, giving a total score range of 0–30. Higher scores reflect a higher frequency and degree of distress. Linley-Adams et al. found the internal consistency of BOA-C to be high (α = 0.81) [22].

Discharge summaries and clinical notes were screened for any psychiatrist diagnoses of a post-stroke mood disorder, including PSD, PSA, or adjustment disorder, as the reference standard.

The information regarding the patients’ clinical outcomes at the time point most proximal to 1-year post-stroke was extracted and described. There were no standardised measures that were consistently documented pre- and post-discharge. Hence, a patient was recorded to have a ‘poor outcome’ if they require more than minimal assistance in their basic Activities of Daily Living (bADLs), are living in a nursing home, or are deceased. A patient’s clinical outcome is ‘not poor’ if they do not require a full-time caregiver and are not deceased. While efforts were made to collect the outcome status nearest to 1-year post-stroke, it was not possible to assess clinical outcomes at more than 8 months post-stroke for 21 (8.9%) out of 236 patients due to the inability to access out-of-hospital medical records or unclear documentation at 1-year post-stroke. Hence, they were excluded from the outcome analysis. The demographics and stroke characteristics were compared between those with and without poor outcomes, and continuous and categorical variables were analysed with a student’s *t*-test and chi-squared test respectively. The level of significance was set at <0.05. Univariate and multivariate logistic regression analyses were carried out thereafter to investigate the potential predictors of a poor outcome.

Data analysis was carried out with the Statistical Package for Social Sciences (SPSS) for Macintosh HD Version 30.0.0.0 (172) (SPSS Inc., Chicago, IL, USA). For this study, a documented psychiatrist’s diagnosis of a post-stroke mood disorder was the reference standard as there is no gold standard diagnostic method for aphasic stroke patients due to the inability to carry out and complete conventional clinical interviews. A psychiatrist’s clinical diagnosis incorporates clinical observation, multiple sources of information from family members and healthcare staff with interactions in different contexts, and the interpretation of pertinent clinical information such as neuroimaging, which is consistent with standard clinical practice when providing care for patients with communication disabilities. Receiver Operating Characteristic curves (ROC) and corresponding the Area Under Curve (AUC) were generated for the SODS, SADQH-10, and BOA-C. The sensitivity, specificity, positive predictive values, and negative predictive values were generated and compared. Cronbach’s alpha for internal consistency was also computed for each tool.

To determine the optimal threshold that separates a ‘positive’ and a ‘negative’ test result, a ROC curve was plotted with sensitivity (true positive rate) as the y-axis, against 1-specificity (false-positive rate) on the x-axis using the total scores of the tool of interest against the psychiatrist’s diagnosis. The AUC is a comprehensive measure of a test’s ability to differentiate the presence or absence of a condition [23]. The closer an AUC is to 1.0, the more superior the test.

The sensitivity of a test represents its ability to identify people with a condition accurately as ‘positive’, while the specificity of a test represents its ability to identify people without a condition accurately as ‘negative’. Positive predictive value (PPV) represents the probability of a person identified as ‘positive’ to have the condition, while negative predictive value (NPV) represents the probability of a person identified as ‘negative’ to not have the condition [24]. The Youden index (*J*) is a function of sensitivity and specificity that ranges from 0 to 1, with an index closer to 1 indicating a relatively better test efficacy [25].

## 3. Results

Table 1 shows the demographics and clinical characteristics of the 236 patients reviewed. The mean age is 63.71 years old and most patients were male and Chinese. Most of the patients were married and less than half were unemployed. The most common stroke characteristics were that of left, non-first, ischaemic stroke, with a large-vessel disease mechanism, of anterior circulation and affecting the basal ganglia.

### 3.1. Performance of SODS

**Figure 2:**
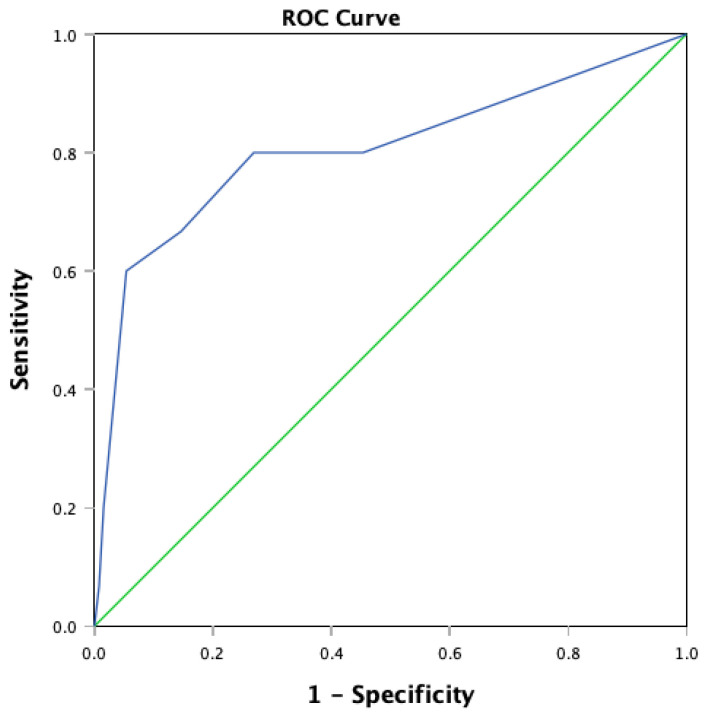
ROC curve of SODS.

The AUC for the SODS is 0.805, CI 0.659–0.950, *p* < 0.001 (Figure 2). A cut-off score of 4 and above has the optimal sensitivity, specificity, PPV, NPV and Youden Index in the study sample (Table 2). The internal consistency was acceptable at Cronbach’s alpha of 0.73 (CI 0.65–0.79). However, specificity (94.6%) and NPV (95.4%) were much better than the sensitivity (60.0%) and PPV (56.3%).

### 3.2. Performance of SADQH-10

**Figure 3:**
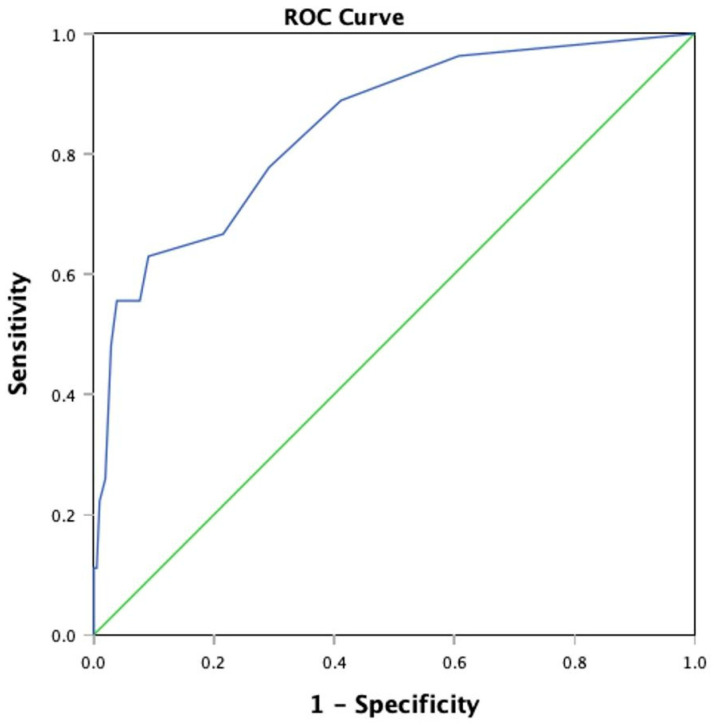
ROC curve of SADQH-10.

The AUC for the SADQH-10 is 0.844, CI 0.763–0.926, *p* < 0.001 (Figure 3). A cut-off score of 7 and above gives the optimal sensitivity, specificity and NPV, without much compromise to the Youden Index (Table 2). The internal consistency was acceptable at Cronbach’s alpha of 0.727 (CI 0.67–0.78). Like the SODS, the specificity (96.2%) and NPV (94.4%) were much better than the sensitivity (55.6%) and PPV (65.2%).

### 3.3. Performance of BOA-C

The AUC for the BOA-C is 0.780, 0.678–0.881, *p* < 0.001. There was no identifiable optimal cut-off threshold of score in relation to a clinician’s diagnosis of mood disorder, as for each cut-off score, PPV and the Youden Index were poor, with none reaching 0.5 (Table 2). The internal consistency was acceptable at Cronbach’s alpha of 0.74 (CI 0.68–0.79).

### 3.4. Clinical Prevalence and Outcomes

From Table 1, out of 236 aphasic post-stroke patients, 27 (11.4%) of them were diagnosed with a post-stroke mood disorder by a psychiatrist. A total of 89 patients (41.4%) did not have a poor outcome, having regained a basic level of function 1-year post-stroke, as they are able to complete their bADLs, such as toileting, showering, feeding, ambulating, independently or with minimum assistance from their carers. A total of 103 (47.9%) patients required more than minimum assistance, either having a full-time carer or living in a nursing home. The remaining 23 (10.7%) patients were deceased. Hence, 58.6% of patients had poor clinical outcomes. From Table 3, patients with poor outcomes are more likely to be older (65.62 ± 13.02 vs. 60.78 ± 11.29, *p* = 0.005), female (42.9% vs. 25.8%, *p* = 0.01), unemployed (52.8% vs. 31.0%, *p* < 0.001), and suffer from a non-first (61.1% vs. 46.1%, *p* = 0.05), non-left-sided (26.2% vs. 13.5%, *p* = 0.03), haemorrhagic stroke (38.1% vs. 30.3%, *p* = 0.04).

Univariate binary logistic regression identified statistically significant unadjusted odds ratios for age, sex, occupation, stroke laterality and incidence. After adjustment with multivariate binary logistic regression, recurrent stroke was found to be predictive of poor outcome with an adjusted odds ratio (AOR) of 2.82 (*p* = 0.03, 95% C.I. 1.135–7.006). Haemorrhagic stroke was also found to be predictive of a poor outcome, with an AOR of 2.29 (*p* = 0.03, 95% C.I. 1.093–4.800).

## 4. Discussion

Optimal cut-off scores were identified for the SODS and SADQH-10 in the detection of post-stroke mood disorder, which had an acceptable Youden Index of more than 0.50 [26]. However, it is important to note that the corresponding NPV and specificity of the chosen cut-off scores are high, while the sensitivity is modest at 55.56% for SODS and 60% for SADQH-10. A test with low sensitivity but high specificity implies that it is not useful for ruling out a disease due to its high false-negative rate and hence is not an ideal screening instrument. However, at the threshold scores identified from our study, the Positive Likelihood Ratio (LR+) is 12 for the SODS and 14 for the SADQH-10, and the Negative Likelihood Ratio (LR-) is 0.42 for the SODS and 0.46 for the SADQH-10. Using Baye’s nomogram [27] and a pre-test probability based on the clinician-diagnosed prevalence of PSD in our study population, this means that a patient who screens positive on either tool has an estimated 60% chance of having PSD, whereas a negative screen means the patient has an estimated <5% chance of having PSD. A higher pre-test probability due to the presence of specific baseline factors such as a history of depression or strokes in strategic locations correlated with depression will result in a post-test probability of 70–80% in the case of a positive test and in the range of 10% in the case of a negative test, using the same LR+ and LR- values. On this basis, we conclude that the SODS and SADQH-10 are still useful tools in our setting, with the caveat that a positive screen on either of these tools necessitates an in-depth clinical assessment by a trained healthcare professional for diagnostic clarification. A negative screen, on the other hand, will need to be repeated at a later time especially if there is clinical suspicion or there are baseline risk factors in a patient. This is the current clinical practice at our site. While a lower-than-expected prevalence of clinician-diagnosed mood disorder in our study raises concerns of under-detection and case ascertainment bias, our clinical experience shows that previously reported rates may be an overestimation due to the lack of distinguishing depression from confounding neurological sequelae such as apathy, which occurs frequently and independently or concurrently with depression [28]. This is the basis for our recommendation for a careful clinical assessment by an experienced clinician in those who screen positive, as this has a direct impact on treatment decisions.

The identified cut-off score of 4 on the SODS was two points greater than the cut-off score of 1/2 (sensitivity of 0.86 and specificity of 0.62) when validated against the Hospital Anxiety and Depression Scale (HADS) by Bennett et al. [21], and one point greater than the cut-off score of 3 (sensitivity of 0.90 and specificity of 0.72) validated against a Geriatric Mental State-AGECAT diagnosis of depression by Hammond et al. [29]. The optimal cut-off score chosen for the SADQH-10 was also one point greater than the cut-off score of 5/6 (sensitivity of 1 and specificity of 0.78) recommended by Bennett et al. when validated against the HADS [21]. The lack of agreement of optimal threshold scores not only indicate the heterogeneity of validation methods but also lends an impetus to the need for appropriate validation in specific clinical populations. Hence, results from our study are a crucial step towards advancing clinical care for aphasic stroke patients in Singapore but are unlikely to be generalizable to other stroke populations in other practice settings.

There was no optimal cut-off score found for the BOA-C in this study. In an earlier study that examined the psychometric properties of the BOA-C in an aphasic stroke population, Eccles et al. recommended the optimal cut-off score of 17 or greater with a sensitivity of 0.85 and specificity of 0.85 against HADS-A [30]. Our results suggests that anxiety symptoms are much less prominent in the phenomenology of PSD in aphasic stroke patients in contrast with what was hypothesised a priori. The lack thereof of a cut-off score limits the utility of the BOA-C in identifying post-stroke mood disorders in our study population.

Associations were identified between poor clinical outcomes and six variables in this preliminary analysis. Older age, female gender, unemployment, having previous stroke and haemorrhagic strokes lead to poor mobility and outcomes, as supported by the literature [31,32,33,34,35]. Poorer outcomes were also associated with non-left-sided strokes in this study, while previous studies have shown conflicting results regarding stroke laterality affecting prognosis and outcome. Stroke type and stroke in strategic locations were not found to be associated with the clinical outcome, contrary to the literature, where stroke lesion location is known to significantly influence aphasia recovery [36] and functional impairment, and patients with large-vessel strokes had the highest rates of mortality and poorer functional outcomes [37]. Further analyses by logistic regression identified recurrent stroke and haemorrhagic stroke to be significant predictors of poor outcomes. Importantly, depression status as defined by reference standard diagnosis was also counter-intuitively not predictive of a poor outcome and is likely due to the inadequate power of the study.

The strengths of the study include its sample size, which is the largest so far among existing similar validation studies. It is also a representative sample of consecutive hospitalised stroke patients in a national stroke centre with the different stroke types, locations, and aetiologies that cause aphasia.

Limitations of this study include the heterogeneity of the sample in terms of stroke type and aphasia type, and the lack of standardised criteria for selecting the carer who would be the most appropriate to rate the aphasic tools. There was also the lack of inter-rater blinding. While these factors may limit the validity of the study results, which does not make them ideal for clinical use, the results are, however, consistent with and reflective of our clinical experience in working with this particular patient population.

Even though aphasic severity was not systematically and routinely assessed, our team would first use their clinical judgement to assess if the patients could give valid self-reports about their mood. If they could not, they would discuss with the attending clinician and sometimes allow the patients more time to see if their communication impairments would improve, before deciding to proceed with the aphasic tools.

Consistency in the reference standard diagnosis was maintained by having the same clinician perform the diagnostic assessment for all the patients in the study, despite not using a structured diagnostic interview.

This study also included patients with prior psychiatric history and patients with antidepressants initiated before the administration of aphasic screening tools which may introduce selection bias. These patients were not excluded from the study population due to their small numbers of 3 and 4, respectively. PSD can still be present at and potentially associated with stroke outcome at 18 months post-stroke [3] and it would be beneficial to conduct a separate validation study for patients after discharge from hospital to evaluate the validity of the tools after the acute phase of stroke. This study was unable to do this due to the small sample size from high drop-out rates from the screening service.

## 5. Conclusions

In conclusion, this study suggests that the SODS and SADQH-10 are appropriate carer-rated screening tools for assessing post-stroke depression in acute stroke patients with aphasia. We recommend that patients who screen positive on either of these tools be referred to an experienced clinician for diagnostic clarification. On the other hand, the BOA-C was not found to be appropriate for screening for post-stroke mood disorder in aphasic patients, and further validation for specific post-stroke anxiety screening is required. More than half of the patients had a poor clinical outcome at 1 year post-stroke, either requiring a full-time home caregiver or institutionalisation, or they were deceased. Recurrent and haemorrhagic strokes were found to be significant predictors of poor clinical outcome. This adds to the imperative need to identify new therapeutic targets or treatments for survivors of aphasic stroke, and whether PSD is one such potential target requires further study.

## Figures and Tables

**Table 1:** Baseline characteristics of aphasic stroke patients reviewed.

	Characteristic	*N*	Mean (SD) or %		Range	
	Age	236	63.71 (12.528)	31.00	–	94.00
**Sex**						
	Female	81	34.3%			
	Male	155	65.7%			
**Race**						
	Chinese	186	78.8%			
	Malay	36	15.3%			
	Others	14	5.9%			
**Marital Status**						
	Married	169	71.6%			
	Single	67	28.4%			
**Highest Education Level**						
	Secondary and below	45	19.0%			
	Tertiary	29	12.3%			
**Occupation**						
	Unemployed	104	44.1%			
	Employed	127	53.8%			
**Stroke Laterality**						
	Left	185	78.4%			
	Right	23	9.7%			
	Bilateral	28	11.9%			
**Stroke Incidence**						
	First Stroke	105	44.5%			
	First Clinical	75	31.8%			
	Recurrent	56	23.7%			
**Stroke Nature**						
	Ischaemic	151	64.0%			
	Haemorrhagic	81	34.3%			
	Ischaemic and Haemorrhagic	4	1.7%			
**Stroke Type**						
	Small-Vessel Disease	85	36.0%			
	Large-Vessel Disease	69	29.2%			
	Others	82	34.7%			
**Stroke Circulation**						
	Anterior	180	76.3%			
	Posterior	28	11.9%			
	Anterior and Posterior	28	11.9%			
**Stroke Structure**						
	Basal Ganglia	114	48.3%			
	Thalamus	20	8.5%			
	Both Basal Ganglia and Thalamus or Others	102	43.2%			
**Clinical Outcome**						
	Not Poor	89	41.4%			
	Full-time Caregiver	103	47.9%			
	Deceased	23	10.7%			
**Variables of Interest**						
	Clinician-diagnosed Mood Disorder	27	11.4%			
	SODS	145	1.17 (1.529)	0	–	6
	SADQH-10	236	2.40 (3.106)	0	–	18
	BOA-C	206	3.12 (3.801)	0	–	19

**Table 2:** Accuracy of screening tools across cut-off scores.

Rating Tool	Threshold Score	Sensitivity	Specificity	PPV	NPV	Youden Index (*J*)
SODS	1	80.00%	54.62%	16.90%	95.95%	0.35
	2	80.00%	73.08%	25.53%	96.94%	0.53
	3	66.67%	85.38%	34.48%	95.69%	0.52
	**4**	**60.00%**	**94.62%**	**56.25%**	**95.35%**	**0.55**
	5	20.00%	98.46%	60.00%	91.43%	0.18
SADQH-10	4	66.67%	78.47%	28.57%	94.80%	0.45
	5	62.96%	90.91%	47.22%	95.00%	0.54
	6	55.56%	92.34%	48.39%	94.15%	0.48
	**7**	**55.56%**	**96.17%**	**65.22%**	**94.37%**	**0.52**
	8	48.15%	97.13%	68.42%	93.55%	0.45
	9	37.04%	96.61%	66.67%	92.31%	0.35
BOA-C	2	84.00%	48.62%	18.42%	95.65%	0.33
	3	80.00%	62.98%	22.99%	95.80%	0.43
	4	72.00%	70.72%	25.35%	84.81%	0.43
	5	64.00%	78.45%	29.09%	94.04%	0.42
	6	48.00%	82.87%	27.91%	92.02%	0.31

**Table 3:** Demographics and stroke characteristics stratified by clinical outcome, unadjusted and adjusted odds ratios after logistic regression for poor outcome.

	**Factors**	**Not Poor Outcome (N = 89)**			**Poor** **Outcome (N = 126)**		
		**Mean (SD) or N (%)**			**Mean (SD) or N (%)**		***p*-Value**
**Demographics**							
	Age	60.78 (11.285)			65.62 (13.016)		0.005 *
Sex							0.01 *
	Female	23 (25.8%)			54 (42.9%)		
	Male	66 (74.2%)			72 (57.1%)		
Race							0.24
	Chinese	69 (77.5%)			101 (80.2%)		
	Malay	12 (13.5%)			21 (16.7%)		
	Others	8 (8.9%)			4 (3.2%)		
Marital Status							0.33
	Married	61 (68.5%)			90 (71.4%)		
	Single	28 (31.5%)			36 (28.6%)		
Highest Education Level							0.35
	Secondary and below	19 (59.4%)			22 (62.9%)		
	Tertiary	13 (40.6%)			13 (37.1%)		
Occupation							<0.001 *
	Unemployed	27 (31.0%)			65 (52.8%)		
	Employed	60 (69.0%)			58 (47.2%)		
**Stroke Characteristics**							
Stroke Laterality							0.03 *
	Left	77 (86.5%)			93 (73.8%)		
	Right	3 (3.4%)			17 (13.5%)		
	Bilateral	9 (10.1%)			16 (12.7%)		
Stroke Incidence							0.05 *
	First Stroke	48 (53.9%)			49 (38.9%)		
	First Clinical	27 (30.3%)			42 (33.3%)		
	Recurrent	14 (15.7%)			35 (27.8)		
Stroke Nature							0.04 *
	Ischaemic	58 (65.2%)			78 (61.9%)		
	Haemorrhagic	27 (30.3%)			48 (38.1%)		
	Ischaemic and Haemorrhagic	4 (4.5%)			0 (0.0%)		
Stroke Type							0.27
	Small-Vessel Disease	26 (29.2%)			51 (40.5%)		
	Large-Vessel Disease	31 (34.8%)			32 (25.4%)		
	Others	32 (36.0%)			43 (34.1%)		
Stroke Circulation							0.11
	Anterior	66 (74.2%)			100 (79.4%)		
	Posterior	8 (9.0%)			16 (12.7%)		
	Anterior and Posterior	15 (16.9%)			10 (7.9%)		
Stroke Structure							0.76
	Basal Ganglia	42 (47.2%)			62 (49.2%)		
	Thalamus	9 (10.1%)			8 (6.3%)		
	Both Basal Ganglia and Thalamus or Others	38 (42.7%)			56 (44.4%)		
**Clinician’s Diagnosis**							
	With Mood Disorder	7 (7.9%)			16 (12.7%)		0.26
	Without Mood Disorder	82 (92.1%)			110 (87.3%)		
	**Predictor**	**Unadjusted OR**			**Adjusted OR**		
		**OR**	**95% CI**	***p*** **-Value**	**OR**	**95% CI**	***p*** **-Value**
	Age	1.032	1.009–1.056	0.01	1.021	0.990–1.053	0.18
Sex							
	Male	1			1		
	Female	2.152	1.191–3.888	0.01	1.852	0.893–3.839	0.10
Occupation							
	Unemployed	1			1		
	Unskilled	0.920	0.424–1.995	0.83	1.484	0.603–3.651	0.39
	Skilled	0.272	0.131–0.566	<0.001	0.451	0.181–1.125	0.09
	Professional/ Executive	0.195	0.075–0.507	<0.001	0.332	0.104–1.060	0.06
Stroke Laterality							
	Left	1			1		
	Right	4.692	1.326–16.606	0.02	2.389	0.620–9.214	0.21
	Bilateral	1.472	0.616–3.516	0.38	1.159	0.399–3.363	0.79
Stroke Incidence							
	First Stroke	1			1		
	First Clinical Stroke	1.524	0.815–2.850	0.19	1.605	0.775–3.327	0. 20
	Recurrent	2.449	1.712–5.115	0.02	**2.820**	**1.135–7.006**	0.03 *
Stroke Nature							
	Ischaemic	1					
	Haemorrhagic	1.322	0.739	0.35	**2.291**	**1.093–4.800**	0.03 *
	Ischaemic and Haemorrhagic	0.000	0.000–NE	1.00	0.000	0.000–NE	1.00

* denotes statistical significance at the 5% level.

## Data Availability

The datasets generated during and analysed during the current study are not publicly available due institution limitations but are available from the corresponding author on reasonable request.

## Abbreviations

The following abbreviations are used in this manuscript:

SODS	Signs of Depression Scale
SADQH-10	Hospital Version of Stroke Aphasic Depression Questionnaire
BOA	Behavioural Outcomes of Anxiety Scale
PSD	Post-Stroke Depression
